# Localization and distribution of goose astrovirus 2 antigens in different tissues at different times

**DOI:** 10.1186/s12917-023-03688-z

**Published:** 2023-09-23

**Authors:** Feng Wei, Xiaoning Jiang, Dalin He, Youxiang Diao, Yi Tang

**Affiliations:** 1 College of Animal Science and Technology, Shandong Agricultural University, 61 Daizong Street, Tai’an, 271018 Shandong Province China; 2 Shandong Provincial Key Laboratory of Animal Biotechnology and Disease Control and Prevention, Tai’an, 271018 Shandong Province China; 3 Shandong Provincial Engineering Technology Research Center of Animal Disease Control and Prevention, Tai’an, 271018 Shandong Province China

**Keywords:** Goose astrovirus 2, Immunohistochemistry, Antigen distribution

## Abstract

Goose astrovirus 2 (GAstV-2) causes visceral gout in goslings and has resulted in significant economic losses in the goose industry of China since its outbreak in 2017. To further investigate the distribution and localization of GAstV-2 in different tissues at different times, a monoclonal antibody (mAb)-based immunohistochemical (IHC) assay was developed to detect GAstV-2. A total of 80 1-day-old healthy goslings were inoculated with GAstV-2 via the oral (n = 40) and intramuscular routes (n = 40). GAstV-2 in the tissues of interest was detected using the established IHC assay. The results showed that positive signals were detected in most tissues at 1 day post-infection (dpi). Viral antigens were mainly distributed in the cytoplasm, and the staining intensity was higher in the renal tubular epithelial cells than in other cells. Taken together, our data demonstrated that GAstV-2 has a broad tissue tropism and primarily targets the kidneys. These results are likely to provide a scientific basis for further elucidation of the pathogenesis of GAstV-2.

## Introduction

Astroviruses (AstVs) are single-stranded, positive-sense and non-enveloped RNA viruses with a whole genome length of 6.4–7.9 kb that belong to the *Astroviridae* family [1]. The AstV genome consists of a 5′-untranslated region (UTR), three open reading frames (ORF1a, ORF1b, and ORF2), a 3′-UTR, and a poly-A tail [2]. Goose astrovirus (GAstV) can be divided into two distinct phylogenetic clades (GAstV-1 and GAstV-2) based on the ORF2 protein [3]. Since 2017, several reports have confirmed that GAstV-2 is one of the culprit pathogens of gosling gout diseases [4–7], which has brought considerable economic losses to the goose industry of China.

Molecular methods for laboratory diagnosis of GAstV-2 infections consist of real-time quantitative reverse transcriptase polymerase chain reaction (qRT-PCR) [8], quantitative loop-mediated isothermal amplification (LAMP) assay [9], immunochromatographic strip assay (ICS) and indirect competitive ELISA assay (ic-ELISA) [10, 11]. qRT-PCR is a sensitive, rapid and specific method for the early detection of GAstV infection and initial diagnosis of the disease. On the other hand, LAMP and ICS are rapid and simple detection methods for on-site diagnosis of the disease. ic-ELISA is a sensitive and specific serological diagnostic method that can be used for immune evaluation and serosurveillance of GAstV-2 infection. However, qRT-PCR, LAMP and ICS are mainly used for the testing of clinically infected samples, while ic-ELISA is primarily utilized for clinical serological diagnosis and immune evaluation. Unlike the above methods, immunohistochemistry (IHC) is an immunological detection method that can qualitatively and quantitatively measure the antigen of interest via antigen-antibody and histochemical reactions. This method not only directly reveals the level of damage caused by the pathogen to the body, but it also unveils the location of the pathogen in various organs, which can provide a basis for understanding the site and mechanism of action of the pathogen [12]. At present, IHC has been extensively used for disease diagnosis and pathogen detection [13–15].

For this reason, we developed a reliable IHC assay to detect GAstV-2 antigens in different tissues at different time points after infection in the hopes of further elucidating the pathogenesis of GAstV-2.

## Materials and methods

### Virus

In 2021, a strain of GAstV-2 named GAstV-SDTZ (GenBank accession number: OP221731) was isolated from goslings suffering from visceral gout (Zaozhuang City, Shandong Province), and was used as the challenge virus for experimental infection. The infectivity titer of the virus was determined to be 10^− 4.9^ TCID_50_/0.1 mL by infection of the chicken liver cell line (ATCC) using the Reed-Muench assay [16]. The challenge virus was free of other waterfowl-dervied viruses (including GAstV-1, H9N2 subtype avian influenza virus, avian orthoreovirus, Tembusu, goose parvovirus, Newcastle disease virus and fowl adenovirus).

### Animal infection

One-day-old healthy goslings were purchased from a commercial hatchery in Jining city, Shandong province, China [17]. A total of 120 one-day-old healthy goslings were divided into three groups (A, B, and C) with 40 goslings per group. The goslings were reared in different negative pressured isolators. Goslings were inoculated with 0.3 mL of viral suspension (GAstV-SDTZ strain; 10^− 4.9^ TCID_50_/0.1 mL) orally in group A and intramuscularly in group B (infected groups). Goslings in group C were orally inoculated with 0.3 mL sterile PBS (control group). Clinical signs and mortality were monitored and recorded daily.

### Sample collection

At 1, 3, 5, 7, 9, 12, 15, 18, 21, and 24 dpi, three goslings were randomly selected from each group and euthanized by bleeding from the jugular vein under anesthesia (30 mg/kg of body weight sodium pentobarbital). Tissue samples, including the liver, spleen, lungs, kidneys, bursa of Fabricius, thymus, pancreas, brain, glandular stomach, and duodenum, were collected from each group for the IHC assay.

### Histopathology

Tissue samples (e.g., liver and kidneys) were collected from each group for histopathology. Tissues were fixed in 10% neutral formalin at room temperature for 48 h, processed and embedded in paraffin, cut into 5 μm paraffin sections, stained with hematoxylin and eosin (H&E), and observed under a light microscope (Nikon, EclipseE100, Japan).

### Immunohistochemistry

Tissue samples were collected from each group for IHC analysis. The tissue sections were dewaxed and then treated with anhydrous ethanol for 3 min, 95% ethanol for 3 min, 85% ethanol for 3 min, 75% ethanol for 3 min and deionized water for 3 min. Antigen retrieval was performed using sodium citrate (pH 6.0) for 7 min under microwave heating, and the tissue sections were then blocked with 5% bovine serum albumin (Beyotime Biotechnology Shanghai, China) for 30 min at 37 °C. Tissue sections were incubated with the primary monoclonal antibody (mAb; 1:1000, prepared by our laboratory as described by Yang et al.) for 12 h at 4 °C [10], incubated with biotinylated goat anti-mouse secondary IgG (1:500; CWBIO, Beijing, China) for 1 h at 37 °C, stained with DAB (Beyotime Biotechnology Shanghai, China) for 5 min, stained with hematoxylin for 2 min, and examined under a light microscope (Nikon, EclipseE100, Japan).

### Specificity of IHC assay

The positive control was the tissue of dead goslings infected with GAstV-2, and the negative control was the tissue of healthy goslings in the control group. The primary antibody was replaced with PBS in the blank test. Mouse anti-goose Tembusu mAb was used as an isotype control for mouse anti-GAstV-2 mAb. The specificity of the IHC assay was determined using liver sections of goslings infected with GAstV-1, H9N2 subtype avian influenza virus, avian orthoreovirus, goose parvovirus, Tembusu, Newcastle disease virus and fowl adenovirus.

### Statistical analysis

Data were analyzed using one-way analysis of variance in GraphPad Prism 8.0 (GraphPad Software Inc.).

## Results

### Clinical signs and gross lesions

**Fig. 1:**
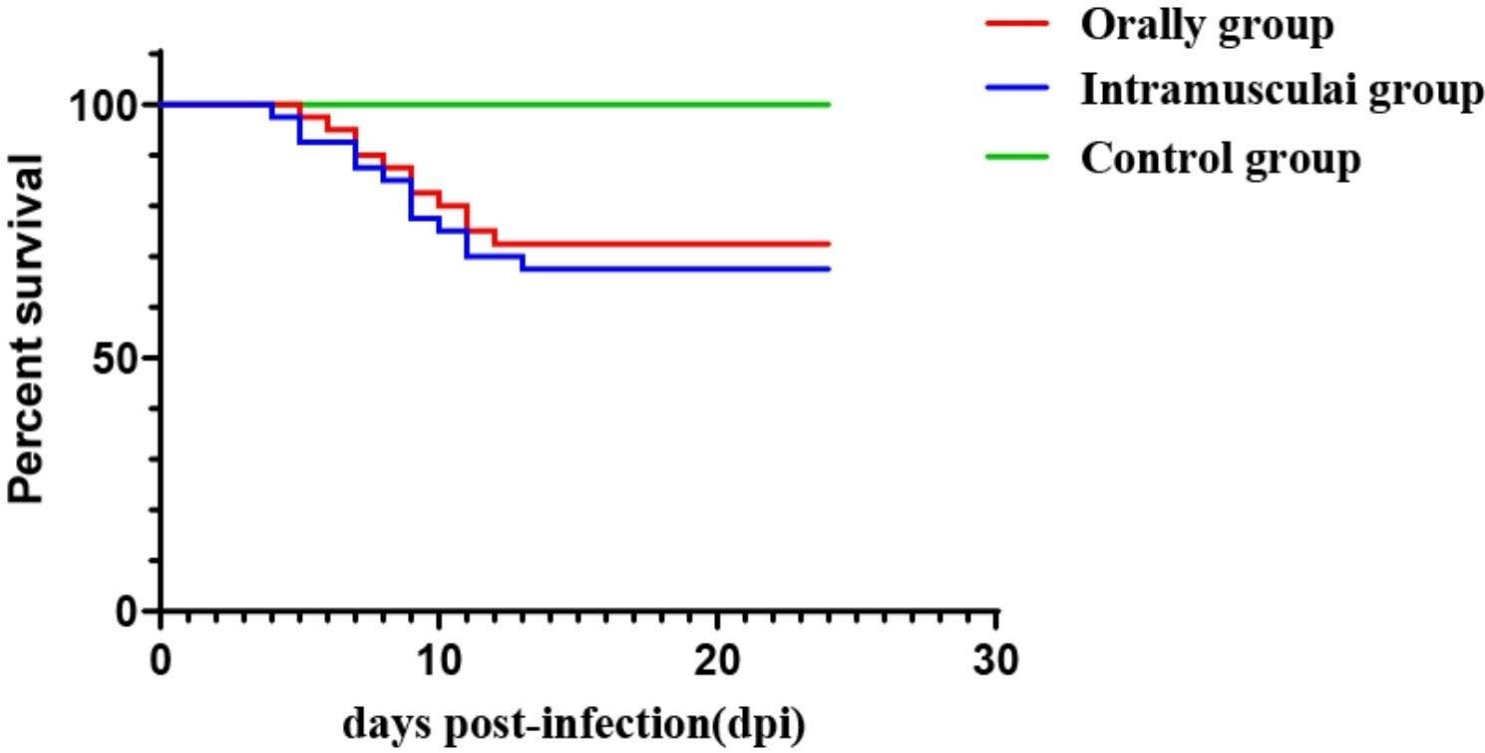
Survival rates of goslings infected with GAstV-SDTZ

In groups A and B, loss of appetite and depression were observed at 3 to 5 dpi. A total of 13 goslings in group A died between 4 and 13 dpi, and 11 goslings in group B died between 5 and 12 dpi (Fig. 1). At necropsy, the kidneys of both infected groups showed severe haemorrhage and swellings.

**Fig. 2:**
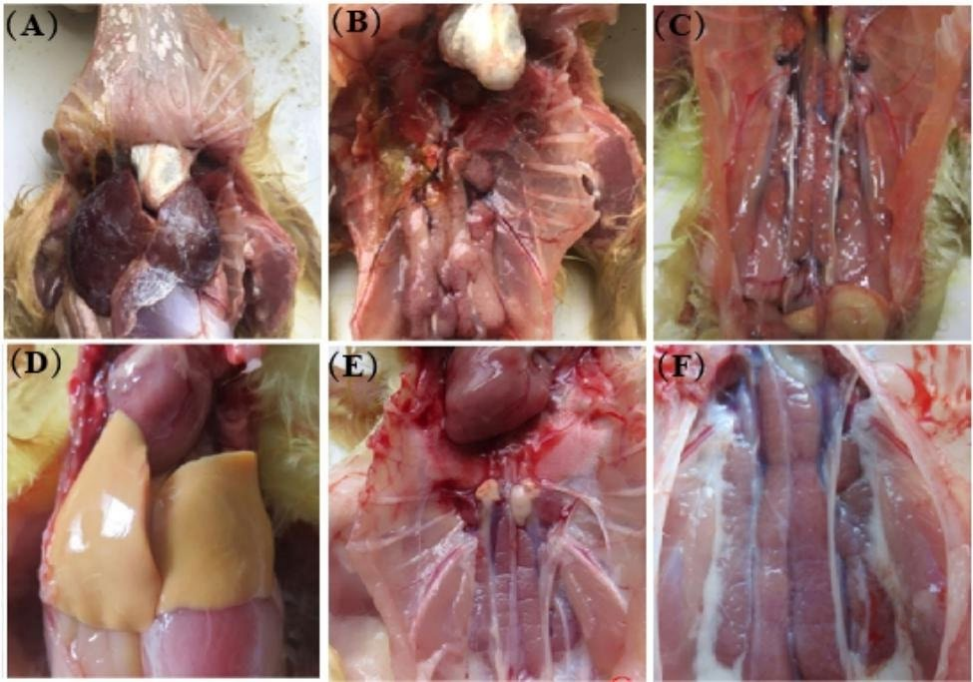
Gross lesions of goslings infected with GAstV-SDTZ. **(A)** Urate deposits on the surface of the liver and heart; **(B-C)** Severe hemorrhage and swelling in the kidneys; **(D-F)** Uninfected goslings

Necropsy revealed significant pathological changes in the kidneys of both infected groups, including severe hemorrhages and renal enlargement. Moreover, gross lesions including urate deposits on the surface of the visceral organs, such as the liver, heart, and kidneys, were observed in dead goslings (Fig. 2). All goslings in group C were healthy during the experimental period.

### Histopathology of different tissues

**Fig. 3:**
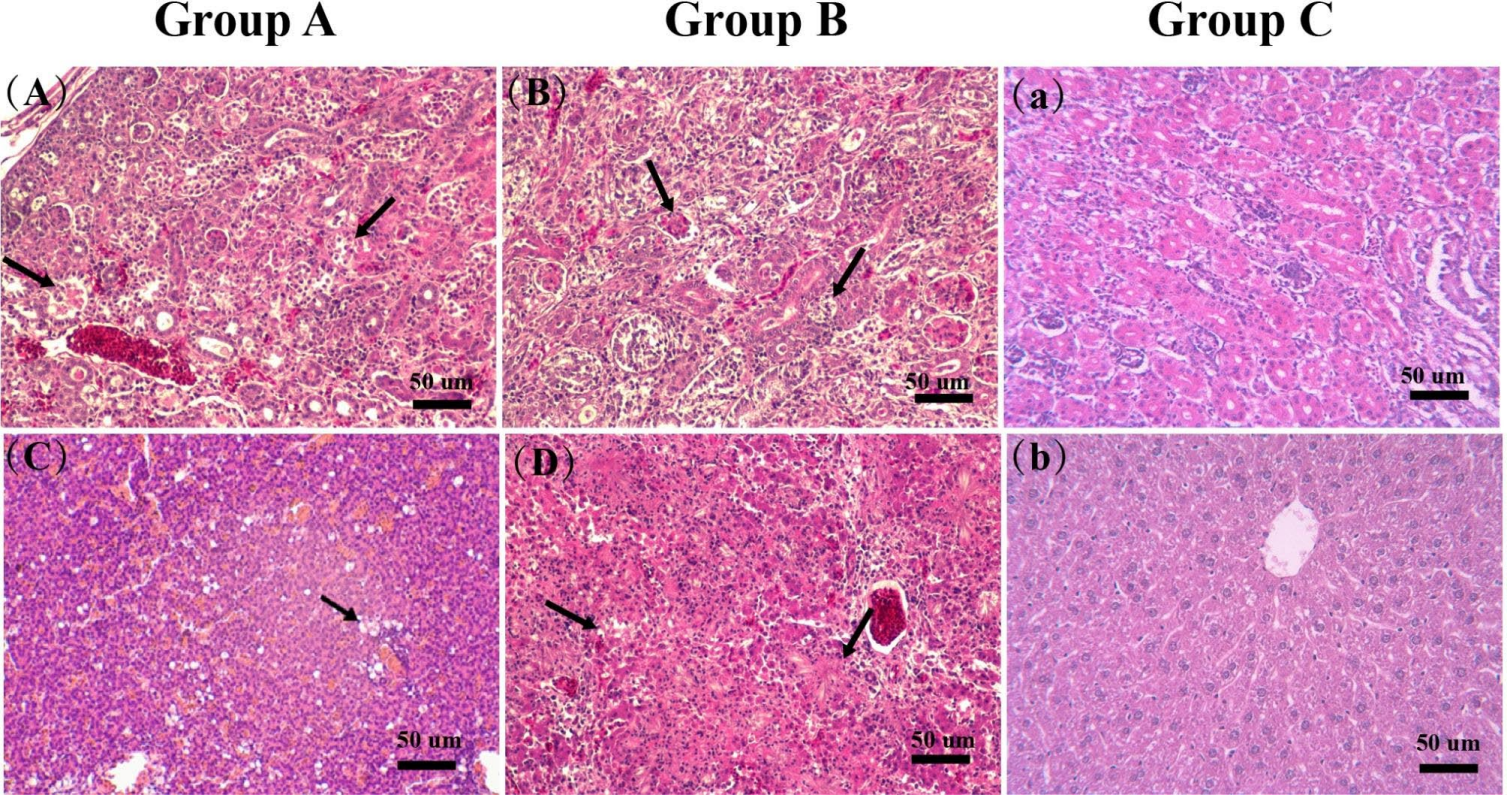
Pathological changes in GAstV-SDTZ-infected goslings. **(A-B)** Renal tubular epithelial cell degeneration, necrosis and exfoliation (black arrows); **(C-D)** Steatosis and infiltration of inflammatory cells in the liver (black arrows). **(a-b)** Uninfected goslings

Histopathological changes were predominantly found in the kidneys and liver. Renal tubular epithelial cell degeneration, necrosis and exfoliation were observed in the kidneys, whereas steatosis, vacuolar degeneration and inflammatory cell infiltration were detected in the liver (Fig. 3A-D). In addition, no microscopic histological lesions were observed in the control group.

### Specificity of the IHC assay

Our results showed that the IHC assay established in this study was only positive for the positive control, and negative for all other control tests.

### Localization and distribution of GAstV-2 in goslings

**Fig. 4:**
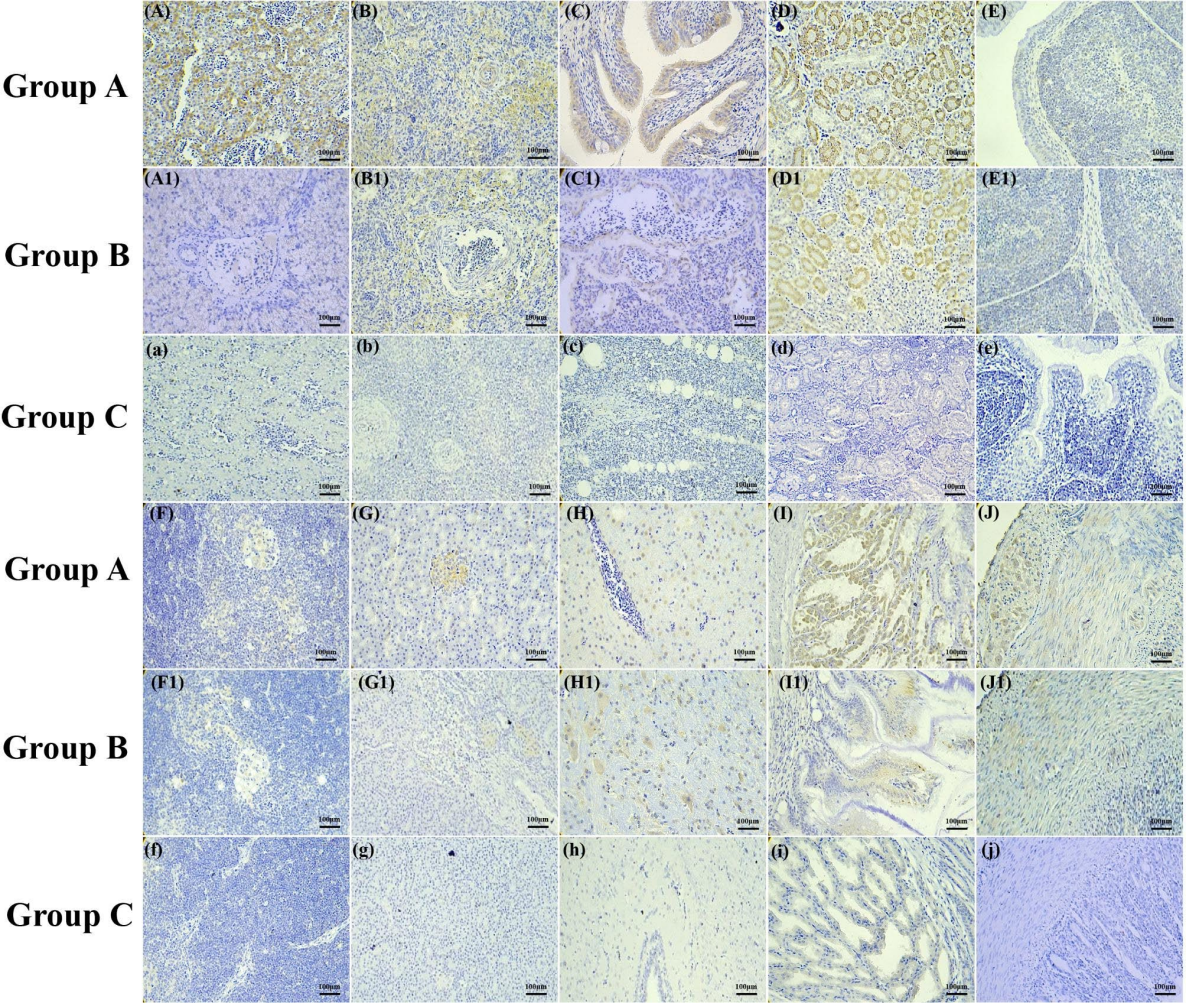
IHC detection of viral antigens in different tissues of goslings after GAstV-SDTZ infection. **(A-A1)** Cytoplasm of hepatocytes, branches of hepatic portal vein and hepatic artery; **(B-B1)** Lymphocytes around the central artery; **(C-C1)** Flat epithelial cells and cuboidal epithelial cells lining the bronchi, respiratory capillary **(D-D1)** Cytoplasm of epithelial cells of proximal convoluted tubules, distal convoluted tubules and collecting ducts; **(E-E1)** Medulla of bursa of Fabricius; **(F-F1)** Cytoplasm of reticular cells in the thymic medulla; **(G-G1)** Cytoplasm of B cells and D cells in pancreatic islets; **(H-H1)** Cytoplasm of a neuron; **(I-I1)** Cytoplasm of glandular cells in the compound duct gland and cytoplasm of columnar epithelial cells in the glandular gastric mucosa; **(J-J1)** Cytoplasm of columnar cells in the duodenal gland, Paneth cell and smooth muscle cells of mucous membrane; **(a-j)** Uninfected goslings

The liver, spleen, lungs, kidneys, bursa of Fabricius, thymus, pancreas, brain, glandular stomach, and duodenum stained positive for GAstV-2 in dead goslings infected with the virus, confirming the broad tissue tropism of GAstV-2 (Fig. 4). Positive signals could be detected in the liver, spleen, lungs, kidneys, thymus, pancreas, glandular stomach, and duodenum at 1 dpi in both infected groups. Positive signals could be detected in the bursa of Fabricius at 3 dpi and in the brain at 5 dpi. Though, positive signal was no longer detectable in the bursa of Fabricius, pancreas, and brain at 21 dpi in both infected groups (Table 1). In addition, positive signals were detected in the glandular stomach and duodenum at 1 dpi in group B and at 3 dpi in group A. Staining intensity was significantly higher from 7 to 12 dpi than on other days, with the highest in the kidneys (Table 2).

**Table 1:** The number of positive cells in different tissues

Tissues	Groups	day post infection(dpi)									
		1	3	5	7	9	12	15	18	21	24
**Liver**	**A**	++	++	+++	+++	+++	+++	+++	+++	++	+
	**B**	++	++	+++	+++	+++	+++	+++	+++	++	+
**Spleen**	**A**	+	+	+	++	++	++	++	++	+	-
	**B**	+	+	+	++	++	++	++	++	+	-
**Lung**	**A**	+	+	+	++	++	++	+	+	+	+
	**B**	+	+	+	++	++	++	+	+	+	+
**Kidney**	**A**	++	+++	+++	+++	+++	+++	+++	+++	++	++
	**B**	++	+++	+++	+++	+++	+++	+++	+++	++	++
**Bursa of fabricius**	**A**	-	+	+	+	+	+	+	+	-	-
	**B**	-	+	+	+	+	+	+	+	-	-
**Thymus**	**A**	+	+	+	+	+	+	+	+	+	-
	**B**	+	+	+	+	+	+	+	+	+	-
**Pancreas**	**A**	+	+	+	+	+	+	+	+	-	-
	**B**	+	+	+	+	+	+	+	+	-	-
**Brain**	**A**	-	-	-	+	+	+	+	-	-	-
	**B**	-	-	-	+	+	+	+	-	-	-
**Glandular stomach**	**A**	+	+	++	++	++	++	++	++	+	+
	**B**	-	+	++	++	++	++	++	++	+	+
**Duodenum**	**A**	+	+	++	++	++	++	++	++	+	+
	**B**	-	+	++	++	++	++	++	++	+	+

Number of positive cells: (-), less than 10%; (+), 10–25%; (+ +), 25–50%; (+ + +), more than 50%

**Table 2:** Positive staining intensity of different tissues

Tissues	Groups	day post infection(dpi)									
		1	3	5	7	9	12	15	18	21	24
**Liver**	**A**	++	++	++	+++	+++	+++	++	++	+	+
	**B**	++	++	++	+++	+++	+++	++	++	+	+
**Spleen**	**A**	+	+	+	++	++	++	++	++	+	-
	**B**	+	+	+	++	++	++	++	++	+	-
**Lung**	**A**	+	+	+	++	++	++	+	+	+	+
	**B**	+	+	+	++	++	++	+	+	+	+
**Kidney**	**A**	+++	+++	+++	+++	+++	+++	+++	+++	++	++
	**B**	+++	+++	+++	+++	+++	+++	+++	+++	++	++
**Bursa of fabricius**	**A**	-	+	+	+	+	+	+	+	-	-
	**B**	-	+	+	+	+	+	+	+	-	-
**Thymus**	**A**	+	+	+	+	+	+	+	+	+	-
	**B**	+	+	+	+	+	+	+	+	+	-
**Pancreas**	**A**	+	+	+	+	+	+	+	+	-	-
	**B**	+	+	+	+	+	+	+	+	-	-
**Brain**	**A**	-	-	-	+	+	+	+	-	-	-
	**B**	-	-	-	+	+	+	+	-	-	-
**Glandular stomach**	**A**	+	+	+	++	++	++	++	+	+	+
	**B**	+	+	+	++	++	++	+	+	+	+
**Duodenum**	**A**	+	+	+	++	++	++	+	+	+	+
	**B**	+	+	+	++	++	++	+	+	+	+

Cell stain strength: (-), No staining; (+), light yellow; (+ +), brownish yellow; (+ + +), brown

## Discussion

GAstV-2 associated goose gout has caused huge economic damage to the goose industry of China [6]. Currently, qRT-PCR and LAMP are used for the detection of viral nucleic acids during acute or subclinical infection. Meanwhile, immunological methods based on mAbs against the capsid protein, such as ic-ELISA and ICS, are designed for viral antigen detection and serological diagnosis. However, these assays are unable to indicate the specific cells in which the virus is located and provide a direct visualization of cell degeneration and necrosis caused by viral infection. Therefore, a mAb-based IHC assay was developed in this study to detect the localization and distribution of GAstV-2 antigens in different tissues.

Previous studies have shown that viral nucleic acid can be detected in all investigated tissues of infected goslings [18]. We examined the distribution of the viral antigens in goslings at different times points after GAstV-2 infection using the established IHC assay and found that positive signals were widely present in various cell types, including hepatocytes, lymphocytes surrounding the central splenic artery, squamous and cubic epithelial cells of the bronchus, epithelial cells of proximal convoluted tubules, distal convoluted tubules and collecting tubules of the kidney, reticular structure in the medulla of the thymus lobule, cytoplasm of B cells and D cells in pancreatic islets, neurons, columnar epithelial cells of the glandular gastric mucosa, duodenal and intestinal gland columnar cells, and Paneth cells. This suggests that GAstV-2 has a broad tissue tropism, which is consistent with other recent reports.

The staining intensity was similar between the oral and intramuscular inoculation groups, and was significantly higher in the kidneys than in other tissues, indicating that the kidneys have the highest viral load, followed by the liver. The bursa of Fabricius showed a positive signal at 3 dpi, and the brain showed a positive signal at 7 dpi. However, viral nucleic acid could be detected in the bursa of Fabricius and brain at 2 dpi by qRT-PCR, which may be attributed to the lower sensitivity of the IHC assay for viral antigen during the early stage of infection when the tissue viral load is low. In addition, positive signals were detected earlier in the glandular stomach and duodenum in the oral inoculation group than in the intramuscular inoculation group, which may be associated with the route of infection and the metabolic pathways induced by the virus. Though, further studies are warranted to confirm these findings.

Uric acid is the end product of purine metabolism [19] and is excreted predominantly by multiple urate transporters on renal proximal epithelial cells [20]. Our results revealed that the staining intensity was significantly higher in the proximal convoluted tubules, distal convoluted tubules and collecting tubules of the kidneys than in other tissues, indicating that renal tubular epithelial cells are the main target cells of GAstV-2 and injury of renal proximal epithelial cells may be an important cause of obstructed uric acid excretion and gout.

Taken together, this study demonstrated that GAstV-2 antigen is widely distributed across various tissues at different times points after infection and is predominantly found in the kidneys. These findings may shed new light to the pathogenesis of GAstV-2 gout.

## Data Availability

The data and materials that support the findings of this study are available from the corresponding author, Youxiang Diao: yxdiao@126.com; upon reasonable request.
